# Validation of childhood lupus specific targets: ensuring accurate assessment of disease control in younger, lighter paediatric patients

**DOI:** 10.1093/rheumatology/keaf057

**Published:** 2025-02-06

**Authors:** Chandni Sarker, Andrea L Jorgensen, Kukatharmini Tharmaratnam, Eslam Al-Abadi, Kate Armon, Kathryn Bailey, Marek Bohm, Mary Brennan, Coziana Ciurtin, Janet Gardner-Medwin, Daniel P Hawley, Alison Kinder, Alice Leahy, Gulshan Malik, Zoe McLaren, Elena Moraitis, Ellen Mosley, Athimalaipet V Ramanan, Satyapal Rangaraj, Annie Ratcliffe, Philip Riley, Heather Rostron, Ethan S Sen, Christian M Hedrich, Michael W Beresford, Eve M D Smith

**Affiliations:** Department of Women's & Children's Health, Institute of Life Course and Medical Sciences, University of Liverpool, Liverpool, UK; Department of Health Data Science, Institute of Population Health, University of Liverpool, Liverpool, UK; Department of Health Data Science, Institute of Population Health, University of Liverpool, Liverpool, UK; Department of Rheumatology, Birmingham Children’s Hospital, Birmingham, UK; Department of Paediatric Rheumatology, Cambridge University Hospitals, Cambridge, UK; Department of Paediatric Rheumatology, Oxford University Hospitals NHS Foundation Trust, Oxford, UK; Department of Paediatric Rheumatology, Leeds Children Hospital, Leeds, UK; Department of Paediatric Rheumatology, Royal Hospital for Sick Children, Edinburgh, UK; Centre for Adolescent Rheumatology, University College London, London, UK; Department of Child Health, University of Glasgow, Glasgow, UK; Department of Paediatric Rheumatology, Sheffield Children’s Hospital, Sheffield, UK; Department of Rheumatology, Leicester Children’s Hospital, University Hospitals of Leicester NHS trust, Leicester, UK; Department of Paediatric Rheumatology, Southampton General Hospital, Southampton, UK; Paediatric Rheumatology, Royal Aberdeen Children’s Hospital, Aberdeen, UK; Rheumatology Department, Royal Liverpool and Broadgreen University Hospitals NHS Trust, Liverpool, UK; Department of Paediatric Rheumatology, Great Ormond Street Hospital, London, UK; Department of Paediatrics, Bradford Royal Infirmary, Bradford, UK; Department of Paediatric Rheumatology, University Hospitals Bristol NHS Foundation Trust & Bristol Medical School, University of Bristol, Bristol, UK; Department of Paediatric Rheumatology, Nottingham University Hospitals, Nottingham, UK; Department of Paediatrics, Musgrove Park Hospital, Taunton, UK; Department of Paediatric Rheumatology, Royal Manchester Children’s Hospital, Manchester, UK; Department of Paediatric Rheumatology, Leeds Children Hospital, Leeds, UK; Paediatric Rheumatology, Great North Children’s Hospital & Faculty of Medical Sciences, Newcastle University, Newcastle upon Tyne, UK; Department of Women's & Children's Health, Institute of Life Course and Medical Sciences, University of Liverpool, Liverpool, UK; Department of Paediatric Rheumatology, Alder Hey Children's NHS Foundation Trust Hospital, Liverpool, UK; Department of Women's & Children's Health, Institute of Life Course and Medical Sciences, University of Liverpool, Liverpool, UK; Department of Paediatric Rheumatology, Alder Hey Children's NHS Foundation Trust Hospital, Liverpool, UK; Department of Women's & Children's Health, Institute of Life Course and Medical Sciences, University of Liverpool, Liverpool, UK; Department of Paediatric Rheumatology, Alder Hey Children's NHS Foundation Trust Hospital, Liverpool, UK

**Keywords:** childhood-SLE, cSLE, treat-to-target, T2T, low disease activity, remission

## Abstract

**Objectives:**

To validate novel childhood-onset systemic lupus erythematosus (cSLE) treat-to-target targets including childhood lupus low disease activity state (cLLDAS), cSLE clinical remission on steroids (cCR) and cSLE clinical remission off steroids (cCR-0), as compared with adult-onset SLE (aSLE) targets.

**Methods:**

Attainment of the aforementioned cSLE-specific and aSLE-specific targets (LLDAS, DORIS 2021 Remission) was assessed at each visit in UK JSLE Cohort Study patients. Univariable and multivariable Prentice–Williams–Peterson (PWP) gap-time models investigated the impact of target attainment on new damage and severe flare.

**Results:**

The cohort included 430 cSLE patients. Attainability was comparable between corresponding cSLE and aSLE targets. Achieving cLLDAS (hazard ratio [HR] 0.18 [95% CI: 0.14, 0.23]), cCR (HR 0.18 [0.13, 0.23]) and cCR-0 (HR 0.17 [0.13, 0.23]) reduced the risk of severe flare (all *P < *0.001). Risk of new damage was reduced in those reaching cLLDAS (HR 0.22 [0.11, 0.44]), cCR (HR 0.25 [0.13, 0.49]) and cCR-0 (HR 0.30 [0.15, 0.60]) (all *P < *0.001). Inappropriate attainment of LLDAS and DORIS remission occurred at 35 and 52 visits, respectively, in younger (median age 7.3 and 8.8 years, respectively) and lighter (median weight 26.8 and 37.1 kg, respectively) patients whilst on prednisolone doses that precluded cSLE target attainment (median 0.17 [IQR 0.16–0.24] and 0.13 [IQR 0.11–0.16] mg/kg/day, respectively).

**Conclusions:**

This study validates novel paediatric-specific targets, demonstrating that achieving cLLDAS, cCR and cCR-0 reduces risks of new damage and severe flare, which is comparable to aSLE targets. Using cSLE-specific targets prevents misclassification of disease activity in paediatric patients, enabling more accurate disease control assessments in younger, lighter patients.

## Graphical abstract

**Figure:**
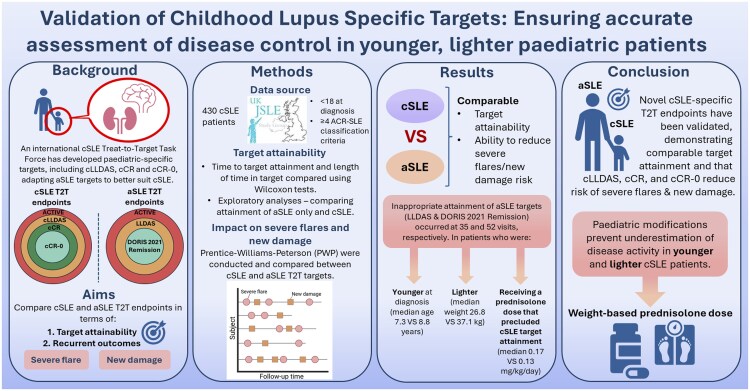

Rheumatology key messages

- cSLE T2T endpoint attainment is associated with reduction in severe flares and new damage risk.
- cSLE and aSLE targets perform comparably in their ability to reduce severe flares/new damage risk.
- Use of cSLE-specific targets prevents underestimation of disease activity in younger, lighter paediatric patients.

## Introduction

Childhood-onset systemic lupus erythematosus (cSLE) is a multisystem autoimmune disease, occurring in individuals ≤18 years of age [1, 2]. The disease has a reported incidence of ∼0.3–0.9 per 100 000 children per year [3]. CSLE is generally more severe than adult-onset SLE (aSLE) at disease onset [4, 5], with a higher prevalence of internal organ involvement compared with aSLE counterparts. Most cSLE patients develop significant damage by early adulthood [6–9], with higher mortality rates [5, 10] and reduced health-related quality of life [11]. Long-term outcomes in cSLE are predominantly influenced by the accumulation of irreversible damage and the frequency of severe flares [12, 13].

The treat-to-target (T2T) approach aims to improve monitoring of individuals with cSLE and aSLE [12, 14], promoting prompt management of disease activity to prevent organ damage and ultimately improve outcomes [15, 16]. Global interest in adopting a T2T approach for both cSLE [17–22] and aSLE [14] has grown. While T2T endpoints in aSLE have been extensively validated, demonstrating improved patient outcomes [23–28], no trials have assessed the value of intervening to achieve T2T targets. The ‘Targeting disease, Agreeing Recommendations and reducing Glucocorticoids through Effective Treatment, in LUPUS’ (TARGET LUPUS©) research programme strives to advance the application of T2T strategies in cSLE [17, 18]. SLE recommendations for T2T in both cSLE [12] and aSLE [14] advocate remission being the main target and low disease activity (LDA) an alternative target. Conceptually, target achievement should deliver protection against severe flares and damage accrual, which are known to worsen long-term disease outcomes [14, 29, 30]. The TARGET LUPUS group has convened an international cSLE T2T task force to establish T2T principles and paediatric-specific targets, including childhood lupus low disease activity state (cLLDAS) [31], cSLE clinical remission on steroids (cCR) and cSLE clinical remission off steroids (cCR-0), endorsed by the Paediatric Rheumatology European Society (PReS) [32].

Paediatric-specific targets have been proposed to improve their applicability to paediatric patients, drawing from validated aSLE targets [29] and incorporating key adjustments, such as weight-based corticosteroid dosing to avoid excessive steroid exposure in cSLE patients [12, 31]. The cSLE T2T task force highlighted the need for two remission targets: one aligned with the aSLE DORIS 2021 criteria, and a second promoting corticosteroid discontinuation, which permits up to 5 mg daily prednisolone [29], to protect growth and reduce long-term damage [32]. This approach ensures safer age-appropriate cSLE targets whilst maintaining consistency for future studies across paediatric and adult populations.

The present study sought to longitudinally evaluate disease outcomes when paediatric-specific T2T endpoints are met, comparing the attainability of novel cSLE targets with the established aSLE targets, and the impact of their attainment in terms of preventing severe flares and new damage, utilizing data from the UK JSLE Cohort Study.

## Methods

### Clinical data

Longitudinal data were collected from the UK JSLE Cohort Study, spanning 2006–2020, across 22 paediatric rheumatology centres [2]. Many UK JSLE Cohort Study centres faced an interruption in data collection during the COVID-19 pandemic. Therefore, we used pre-pandemic data to maintain longitudinal analysis integrity. Participants that were aged ≤18 years at diagnosis and fulfilled ≥4 ACR-1997 SLE classification criteria were included. Written, informed assent/consent was obtained from patients and parents for participation in the UK JSLE Cohort Study. Research was implemented in accordance with the Declaration of Helsinki. Full ethical approval was received from the National Research Ethics Service Northwest (Liverpool, UK, reference 06/Q1502/77).

Clinical data were collected at the time of recruitment to the study, as well as during follow-up, including patient demographics (diagnosis age, gender, ethnicity and disease duration at each visit), cSLE disease activity including the full Systemic Lupus Erythematosus Disease Activity Index-2000 (SLEDAI-2K) score, clinical-SLEDAI-2K score (cSLEDAI, excluding C3, C4 and anti-dsDNA) and the paediatric British Isles Lupus Assessment Grade (pBILAG2004) scores, American College of Rheumatology (ACR) SLE classification criteria, and the Systemic Lupus International Collaborating Clinics Standardized Damage Index (SDI) score, and blood/urine laboratory measurements. Comparisons of paediatric- and adult-specific target definitions are presented in Table 1.

**Table 1.** Comparisons of paediatric-specific and adult-specific T2T target definitions

Paediatric-specific targets [31, 32]	Adult-specific targets [25, 29]
cLLDAS	Original LLDAS
SLEDAI-2K score ≤4 with no major active organ involvement (cardiopulmonary, central nervous system, fever, renal, and vasculitis). No new features of lupus activity compared with previous assessment. Physician global assessment score of ≤1. Prednisolone dose of *0.15 mg/kg/day* or a maximum of 7.5 mg/day and no intravenous methylprednisolone. Tolerated standard maintenance immunosuppressive drugs or biologic agents.*Maintenance treatment considered stable if changes are not due to disease activity, but made due to side effects, adherence, growth and/or when building up to target dose*.	SLEDAI-2K score ≤4 with no major active organ involvement (cardiopulmonary, central nervous system, fever, renal and vasculitis). No new features of lupus activity compared with previous assessment. Physician global assessment score of ≤1. Prednisolone dose of ≤7.5 mg/day and no intravenous methylprednisolone. Tolerated standard maintenance immunosuppressive drugs or biologic agents.
cSLE clinical remission on steroids (cCR)	DORIS 2021 Remission
Clinical SLEDAI score equal to 0. Physician global assessment score of ≤0.5. Prednisolone dose of *0.10 mg/kg/day* or a maximum of 5.0 mg/day and no intravenous methylprednisolone. Tolerated standard maintenance immunosuppressive drugs or biologic agents.*Maintenance treatment considered stable if changes are not due to disease activity, but made due to side effects, adherence, growth and/or when building up to target dose*.	Clinical SLEDAI score equal to 0. Physician global assessment score of ≤0.5. Prednisolone dose of ≤5 mg/day and no intravenous methylprednisolone. Tolerated standard maintenance immunosuppressive drugs or biologic agents.
cSLE clinical remission off steroids (cCR-0)	*NA* *Single definition of remission proposed by the DORIS Task Force since 2021*
Clinical SLEDAI score equal to 0. Physician global assessment score of ≤0.5. No prednisolone. Tolerated standard maintenance immunosuppressive drugs or biologic agents.*Maintenance treatment considered stable if changes are not due to disease activity, but made due to side effects, adherence, growth and/or when building up to target dose*.	

Differences between the cSLE and aSLE specific targets are shown in italics. cCR: cSLE clinical remission on steroids; cCR-0: cSLE clinical remission off steroids; cLLDAS: childhood lupus low disease activity state; DORIS 2021 Remission: single definition of remission proposed by the DORIS Task Force in 2021; LLDAS: original lupus low disease activity state; SLEDAI-2K: Systemic Lupus Erythematosus Disease Activity Index; cSLEDAI; Clinical Systemic Lupus Erythematosus Disease Activity Index; T2T: treat-to-target.

### Statistical analysis

All data analyses were undertaken in R (version 4.3.2) (R Foundation for Statistical Computing, Vienna, Austria) [33].

### Descriptive statistics of demographic and clinical characteristics

Demographic and clinical characteristics were summarized using descriptive statistics. Continuous variables were reported as medians and interquartile range (IQR) as data were skewed. Categorical variables were summarized with frequencies and percentages. Normality was assessed using histograms and the Shapiro–Wilk test, where a *P-*value <0.05 denoted deviation from normality. Results were considered statistically significant if the *P-*value was <0.05.

### Descriptive analyses of attainment of targets

Summary statistics for attainment of the various targets were provided both per patient and per visit. The time-to-target attainment, length and percentage of time in each target were calculated, comparing paediatric- and adult-specific targets using the Wilcoxon signed-rank/rank-sum test. Exploratory analyses compared patients who met adult-specific targets only with those who met the paediatric targets, using the Wilcoxon test to explore parameters such as visit count, age at diagnosis, weight, weight-based prednisolone dosage and PGA. Bonferroni correction was applied for multiple comparisons.

Venn diagrams visualized the number of visits in each target, assessing separation and overlap across target definitions. Reasons for non-attainment of LDA definitions despite reaching a SLEDAI-2K score of ≤4 were explored visually using circular packing plots. Similarly, reasons for non-attainment of remission definitions, despite possessing a cSLEDAI score of 0, were also explored. These plots provide insights into the differences between adult- and paediatric-specific targets, helping to explain instances of misclassification of target attainment.

### Examination of factors associated with spending a greater length of time in target

We examined clinical and demographic factors from the initial visit to identify those associated with spending a greater length of time in target. Time in target was defined as the ratio of time spent in target relative to total follow-up time, with a greater length indicating time above the median. This was computed by dividing the cumulative length of time in target by the total follow-up time. Patients who did not meet the targets, along with those whose cumulative time in target was at or below the median percentage, were categorized together. Univariable logistic regression models (‘glm()’ R function) were implemented, which assessed the impact of individual factors (Supplementary Table S1, available at *Rheumatology* online) on spending a greater length of time in target. Odds ratios (ORs) and 95% CIs were computed for each model.

### Exploration of factors associated with new damage and severe flare

Severe flare was defined as having a pBILAG-2004 score of A or B in any organ domain during patient follow-up [2]. New damage was defined by an increase in the SLICC-SDI score of >1 unit since the last visit. Prentice–Williams–Peterson (PWP) gap-time models effectively explored recurrent events like severe flares and new damage, incorporating ordered multiple events stratified by the number of events during the follow-up period [34]. Univariable PWP gap-time models were fitted (‘coxph()’ function from ‘Survival’ R package), examining the effect of factors (Supplementary Table S1, available at *Rheumatology* online) on new damage and severe flare over time [35]. Factors found significant univariably (*P < *0.05) were included in multivariable PWP gap-time models, with an additional binary covariate representing attainment of target or not at each visit. Multivariable models were not implemented for new damage, as no significant factors were identified from univariable analyses. Martingale and deviance residual plots were conducted to assess model fit, with random scatter around zero indicating a well-fitted model. Hazard ratios (HRs) and 95% CIs were reported. The HRs for target attainment from PWP gap-time models were compared between paediatric- and adult-specific targets using a two-sided Student’s *t*-test for dependent samples. Bonferroni correction was applied to *P*-values to account for multiple comparisons. Impact of cumulative follow-up time spent in target on severe flare was also explored, highlighting time spent in each target from 10% to 80% of the time, which reported HRs.

### Approach to imputation

Imputation of missing weight values at patient visits enabled calculations of weight-based prednisolone dose. Imputation was undertaken in three different ways. It predominantly involved imputation using documented weights from 6 months before or after the visit. When unavailable, we used the 50th centiles of weight from the ‘childsds’ R package for patients up to 18 years, based on WHO growth data [36, 37]. For patients over 18 years, the ‘zoo’ R package was used, performing the ‘last observed carried forward’ technique to impute the last observed weight values [38]. Notably, the imputed weights could only impact target attainment in patients who are <50 kg. If initial visit data were missing, values from within 6 weeks of that visit were used, and these were integrated in logistic regression models to investigate predictors of longer target duration.

## Results

### Clinical and demographic characteristics of patients

Four hundred and thirty UK JSLE Cohort Study participants were included, with a median (IQR) of 10 (5.0–15.0) visits per patient over 2.0 (0.7–4.0) years, with 4738 visits recorded in total. There were 359 females (83.5%) and 71 males (16.5%). The median age at diagnosis was 12.8 (10.4–14.6) years, with a median of 5 (5.0–7.0) ACR criteria fulfilled at diagnosis. This cohort predominantly consisted of 218 White British patients (51%), followed by 129 Asian patients (30%) and 72 African/Caribbean patients (17%). At study recruitment, damage was already present in 66 patients (15%). Damage was documented in 1295/4738 visits during follow-up, accounting for 27% of all follow-up visits. Severe flares were present at 2013/4738 visits during follow-up, constituting 43% of all follow-up visits. The median baseline weight of patients in the cohort was 48.4 (36.5–57.0) kg (Table 2).

**Table 2.** Clinical and demographic characteristics of the patient cohort

Clinical and demographic characteristics	Value
Sex, *n* (%)	
Males	71/430 (16.5)
Females	359/430 (83.5)
Ethnicity, *n* (%)^a^	
White British	218/430 (51.0)
Asian	129/430 (30.0)
African/Caribbean	72/430 (17.0)
Age at diagnosis, median (IQR), years	12.8 (10.4–14.6)
Weight at baseline, median (IQR), kg	48.4 (36.5–57.0)
Length of disease, median (IQR), years	2.0 (0.7–4.0)
Length of time to diagnosis, median (IQR), years	0.3 (0.1–0.9)
Number of visits per patient, median (IQR)	10.0 (5.0–15.0)
ACR criteria at diagnosis, median (IQR)	5.0 (5.0–7.0)
ANA positive at study recruitment, *n* (%)	417/430 (97.0)
Anti-dsDNA positive at any time point, *n* (%)	299/430 (70.0)
SDI score at study recruitment, *n* (%)^b^	
No damage (SDI = 0)	344/430 (80.0)
Mild damage (SDI = 1)	49/430 (11.4)
Moderate damage (SDI = 2)	9/430 (2.1)
Severe damage (SDI ≥ 3)	8/430 (1.9)
SDI score during all follow-up visits, *n* (%)^b^	
No damage (SDI = 0)	3150/4738 (66.5)
Mild damage (SDI = 1)	865/4738 (18.3)
Moderate damage (SDI = 2)	202/4738 (4.3)
Severe damage (SDI ≥ 3)	228/4738 (4.8)
Number of severe flares at study recruitment, *n* (%)	328/430 (76.3)
Number of severe flares during all follow-up visits, *n* (%)	2013/4738 (42.5)
Number of patients reaching target during follow-up (*n* = 430), *n* (%)	
cLLDAS	290 (67)
LLDAS	293 (68)
cCR	249 (58)
DORIS 2021 Remission	261 (61)
cCR-0	202 (47)
Number of visits reaching target during follow-up (*n* = 4738), *n* (%)	
cLLDAS	943 (20)
LLDAS	978 (21)
cCR	796 (17)
DORIS 2021 Remission	848 (18)
cCR-0	595 (13)
Time to target attainment, median (IQR), months	
cLLDAS	18.4 (8.7–31.7)
LLDAS	17.4 (8.1–30.0)
cCR	20.4 (10.3–35.7)
DORIS 2021 Remission	19.3 (9.9–32.7)
cCR-0	23.4 (11.7–36.0)
Percentage of time in target per patient, median (IQR)	
cLLDAS	24.0 (13.0–39.3)
LLDAS	24.0 (12.8–39.1)
cCR	24.1 (12.2–41.1)
DORIS 2021 Remission	24.1 (11.9–42.8)
cCR-0	23.3 (11.4–41.8)
Length of time in target in, median (IQR), months	
cLLDAS	10.3 (6.0–20.5)
LLDAS	10.1 (6.0–21.4)
cCR	10.7 (5.6–21.8)
DORIS 2021 Remission	10.6 (5.5–22.3)
cCR-0	10.4 (5.5–20.5)

Self-reported ethnicity information was collected in accordance with the UK National Census categorizations. Data of mixed-race patients were grouped with associated ethnic minority groups.

a Ethnicity data were not available for 11 patients.

b SDI score at study recruitment was not available for 20 patients. cCR/cCR-0: cSLE clinical remission on/off steroids; cLLDAS: childhood lupus low disease activity state; DORIS 2021 Remission: single definition of remission proposed by the DORIS Task Force in 2021; ACR: American College of Rheumatology; ANA: antinuclear antibodies; anti-dsDNA positive: anti-double stranded DNA antibody positivity; IQR: interquartile range; LLDAS: lupus low disease activity state; SDI: SLICC Standardized Damage Index.

### Target attainment of paediatric-specific *vs* adult-specific targets

**Figure 1.**
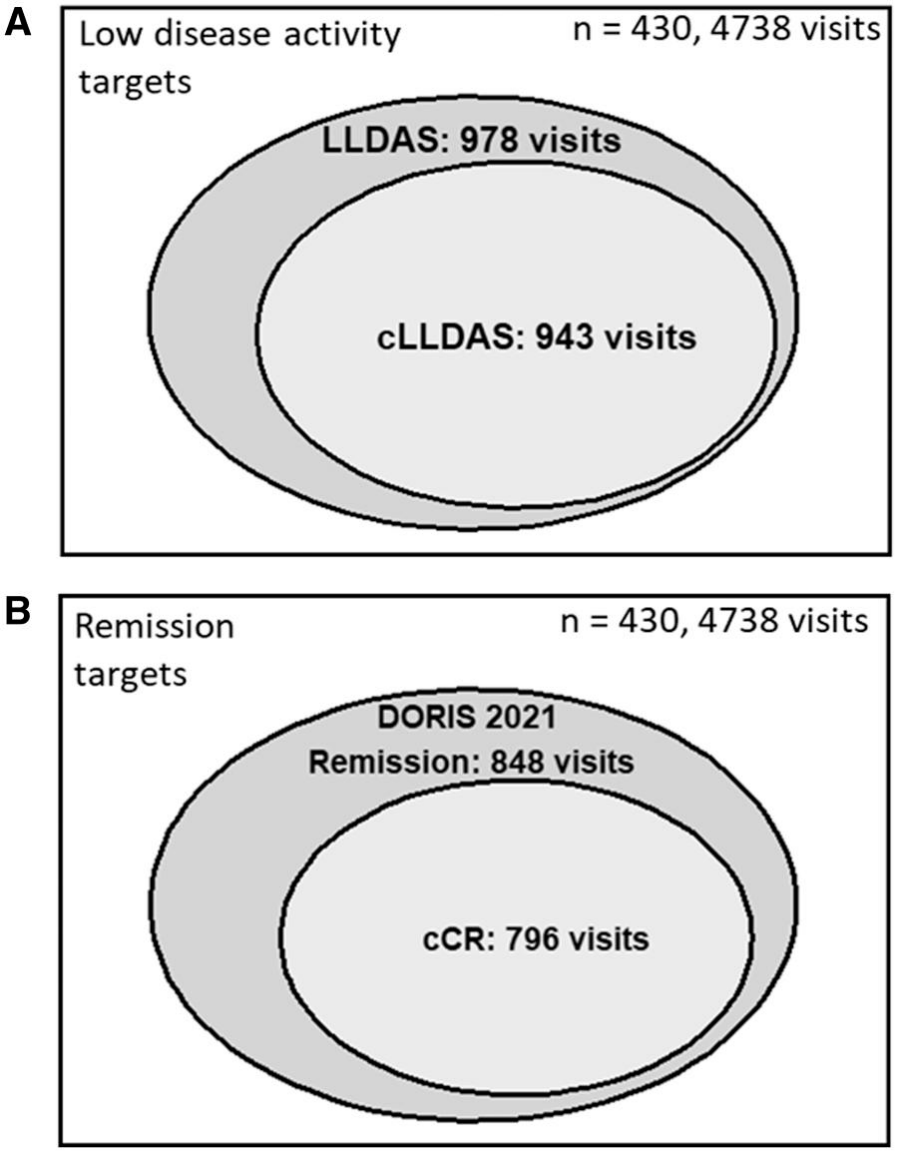
Venn diagrams depicting overlap between attainment of cSLE and aSLE targets. (**A**) Overlap between low disease activity targets (LLDAS and cLLDAS). (**B**) Overlap between remission targets (DORIS 2021 Remission and cCR). aSLE: adult-onset systemic lupus erythematosus; cCR: cSLE clinical remission on steroids; cCR-0: cSLE clinical remission off steroids; cLLDAS: childhood lupus low disease activity state; cSLE: childhood-onset systemic lupus erythematosus; DORIS 2021 Remission: single definition of remission proposed by the DORIS Task Force in 2021; LLDAS: lupus low disease activity state

A comparable number of patients attained cLLDAS (67%) and LLDAS (68%), cCR (58%) and DORIS 2021 Remission (61%). The additional remission target, cCR-0, was attained in 202 (47%) patients at some point during follow-up (Table 2). Reaching paediatric-specific targets took significantly longer statistically than the adult-specific targets, although the time difference was not striking (cLLDAS: 18.4 and LLDAS: 17.4, cCR: 20.4 and DORIS 2021 Remission: 19.3, all *P < *0.03, Supplementary Table S2, available at *Rheumatology* online). Amongst the paediatric-specific targets, attainment of cLLDAS was significantly quicker than cCR and cCR-0 (all *P < *0.02, Supplementary Table S2, available at *Rheumatology* online). Minor (non-significant) differences were denoted in the percentage cumulative time spent in paediatric *vs* adult-specific targets (Table 2, Supplementary Table S2, available at *Rheumatology* online). Figure 1 illustrates the overlap between attainment of paediatric and adult-specific target definitions on a per-visit basis.

### Exploration of patients reaching adult-specific targets but not paediatric-specific targets

There were 35 and 52 visits where adult-specific targets (LLDAS and DORIS 2021 Remission, respectively) were attained, but the corresponding paediatric-specific targets (cLLDAS and cCR, see Fig. 1) were not. At these visits, patients achieving only the adult LLDAS target were significantly younger (7.3 [5.8–8.8] years) compared with those achieving cLLDAS (12.5 [10.3–14.2] years, *P < *0.001). Similarly, patients attaining DORIS 2021 Remission but not cCR were younger (8.8 [7.4–11.7] *vs* 12.3 [10.2–14.0] years, *P < *0.001).

Patients were also significantly lighter at visits where only adult-specific targets were attained. The median weight was 26.8 (20.3–31.4) kg for LLDAS only *vs* 55.2 (45.2–64.2) kg for cLLDAS (all *P < *0.001). For DORIS 2021 Remission *vs* cCR, the median weight was 37.1 (28.2–44.0) *vs* 55.5 (46.6–64.1) kg (all *P < *0.001). At visits where only adult-specific targets were attained, the weight-based prednisolone dose exceeded paediatric-specific target thresholds (LLDAS only: 0.17 [0.16–0.24] mg/kg/day *vs* cLLDAS: 0.08 [0.04–0.10] mg/kg/day and DORIS 2021 Remission: 0.13 [0.11–0.16] mg/kg/day *vs* cCR: 0.06 [0.03–0.08] mg/kg/day, *P < *0.001) (Table 3).

**Table 3.** Comparison of patients reaching adult-specific targets and paediatric-specific targets

	LLDAS only attained	cLLDAS and LLDAS attained	*P*-value, LLDAS only *vs* cLLDAS attained	DORIS 2021 Remission only attained	cCR and DORIS 2021 Remission attained	*P*-value, DORIS 2021 Remission only *vs* cCR attained
Number of visits, *n*	35	943	—	52	796	—
Age at diagnosis, median (IQR), years	7.3 (5.8–8.8)	12.5 (10.3–14.2)	**0.001**	8.8 (7.4–11.7)	12.3 (10.2–14.0)	**0.001**
Weight^a^, median (IQR), kg	26.8 (20.3–31.4)	55.2 (45.2–64.2)	**0.001**	37.1 (28.2–44.0)	55.5 (46.6–64.1)	**0.001**
Weight-based prednisolone dose, median (IQR), mg/kg/day	0.17 (0.16–0.24)	0.08 (0.04–0.10)	**0.001**	0.13 (0.11–0.16)	0.06 (0.03–0.08)	**0.001**
PGA, median (IQR)	0.29 (0.10–0.40)	0.09 (0.00–0.27)	**0.005**	0.10 (0.00–0.30)	0.03 (0.00–0.15)	0.066

Comparisons between adult-specific targets only and paediatric targets using the Wilcoxon test; *P < *0.05 indicated statistical significance, indicated by values in bold. Notably, patients who attained paediatric targets also attained adult-specific targets.

a Weight data are missing for 1249/4738 visits (26%). Of these visits, 466 (9.8%) had a weight of <50 kg, potentially affecting the weight-based prednisolone dosing calculation. aSLE: adult-onset systemic lupus erythematosus; cCR: cSLE clinical remission on steroids; cLLDAS: childhood lupus low disease activity state; cSLE: childhood-onset systemic lupus erythematosus; DORIS 2021 Remission: single definition of remission proposed by the DORIS Task Force in 2021; IQR: interquartile range; PGA: physician global assessment.

### Exploration of the reasons for non-attainment of paediatric and adult-specific targets

**Figure 2.**
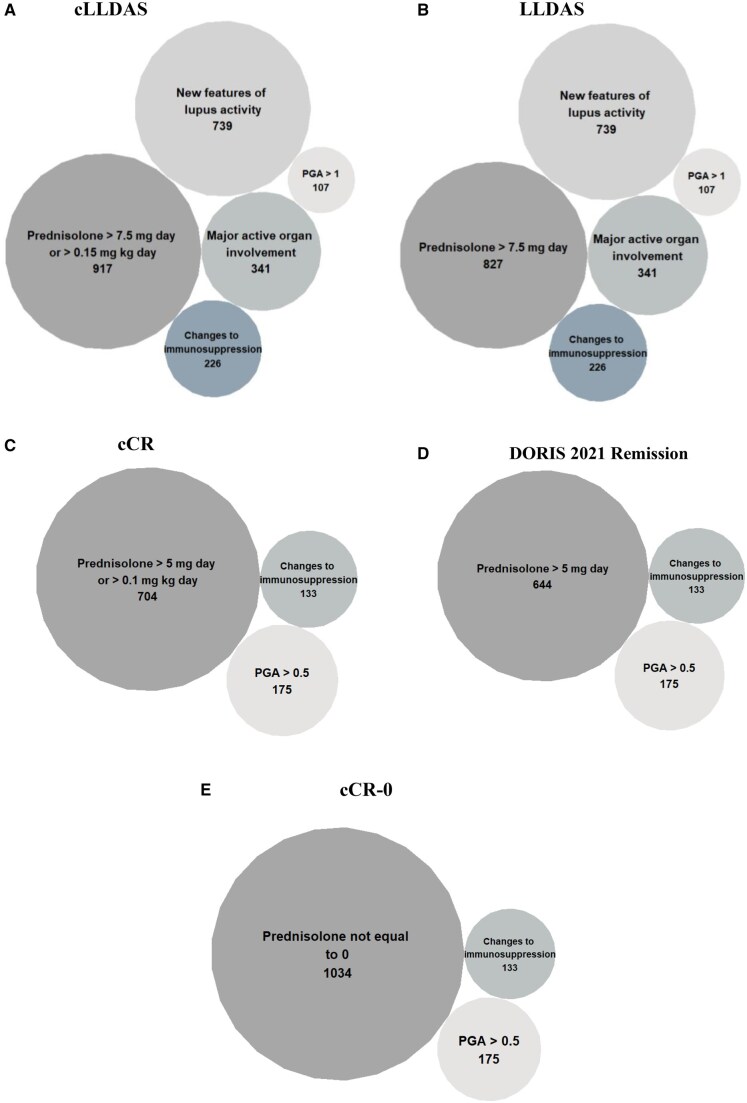
Circular packing plots indicating number of visits for non-attainment of each criterion for each target. (**A**) Non-attainment of cLLDAS despite a SLEDAI-2K score of ≤4. (**B**) Non-attainment of LLDAS despite a SLEDAI-2K score of ≤4. (**C**) Non-attainment of cCR despite the cSLEDAI score being 0. (**D**) Non-attainment of DORIS 2021 Remission despite the cSLEDAI score being 0. (**E**) Non-attainment of cCR-0 despite the cSLEDAI score being 0. cCR: cSLE clinical remission on steroids; cCR-0: cSLE clinical remission off steroids; cLLDAS: childhood lupus low disease activity state; LLDAS: lupus low disease activity state; DORIS 2021 Remission: single definition of remission proposed by the DORIS Task Force in 2021; PGA: physician global assessment

Circular packing plots in Fig. 2 visually represent factors most frequently hindering target attainment across different disease activity and remission definitions. Larger circles indicate more frequent contributors, while smaller circles represent less common contributors. Prednisolone dosage was the main criterion precluding paediatric-specific target attainment as compared with the corresponding adult-specific targets. For example, cLLDAS was precluded due to the patients’ prednisolone dosage exceeding paediatric-specific target thresholds at 917 visits, and LLDAS in 827 visits (Fig. 2A and B). Similarly, cCR was precluded at 704 visits, and DORIS 2021 Remission in 644 visits due to the prednisolone dosage (Fig. 2C and D).

For both cLLDAS and LLDAS, the second most common feature preventing target attainment was new features of lupus activity, followed by presence of significant active organ involvement, changes in immunosuppressive therapy, and lastly, a PGA score exceeding 1. In the remission targets, after the prednisolone dosage criterion, the second most common feature preventing remission targets being met was the PGA score being >0.5, followed by changes in immunosuppressive therapy.

### Predictors of spending a greater length of cumulative time in target

Clinical factors shown to be associated with spending more follow-up time in target univariably are presented in Supplementary Table S3, available at *Rheumatology* online. Low C3 levels were consistently associated with spending less follow-up time in all the paediatric- and adult-specific targets (all ORs <1.00, all *P < *0.05). Low C4 levels were shown to be associated with spending less follow-up time in cLLDAS, LLDAS, cCR and DORIS 2021 Remission (all ORs <1.00, all *P < *0.05). Anti-dsDNA positivity was associated with spending less follow-up time in cLLDAS (OR: 0.41; 95% CI: 0.24, 0.70; *P < *0.05) and LLDAS (OR: 0.38; 95% CI: 0.22, 0.64; *P < *0.001). Being of Asian ethnicity was associated with spending a greater length of time in LLDAS compared with African Caribbean patients (OR: 2.01; 95% CI: 1.02, 3.97; *P < *0.05). Male sex was associated with spending greater follow-up time in cCR, DORIS 2021 Remission and cCR-0 (all ORs >1.00, all *P < *0.05). Other parameters shown to be associated with spending less time in cCR-0 included lymphopenia and older age at diagnosis (all ORs <1.00, all *P < *0.05).

### Impact of target attainment on hazard of having a subsequent severe flare

Univariable analysis demonstrated an association between the following factors and reduced hazards of severe flare; disease duration over 1 year (HR: 0.80; 95% CI: 0.74, 0.86; *P < *0.001) and being of White British or Asian ethnicity compared with African/Caribbean (HR: 0.78; 95% CI: 0.62, 0.98; *P < *0.05 and HR: 0.79; 95% CI: 0.64, 0.97; *P < *0.05, respectively), or attaining any of the target states: cLLDAS, LLDAS, cCR, DORIS 2021 Remission or cCR-0 (HR between 0.15 and 0.17 according to the target state, all *P < *0.001). Conversely, having damage at study recruitment and an increasing SDI score during follow-up was associated with increased hazards of subsequent severe flare (HR: 1.13; 95% CI: 1.02, 1.25; *P < *0.05 and HR: 1.10; 95% CI: 1.04, 1.17; *P < *0.001, respectively) (Supplementary Table S4, available at *Rheumatology* online).

Supplementary Table S5, available at *Rheumatology* online, displays the HRs for having a severe flare with different cumulative follow-up time spent in target. For instance, extending the time in cLLDAS target from 10% to 80% of the follow-up period decreases the risk of severe flare from an HR of 0.69 to 0.05. The same pattern was seen for all other targets.

### Multivariable PWP gap-time models assessing severe flare

Multivariable PWP gap-time models assessing impact on severe flares, demonstrated that meeting any of the LDA target definitions (cLLDAS HR: 0.18; 95% CI: 0.14, 0.23; *P < *0.001; LLDAS HR: 0.18; 95% CI: 0.14, 0.24; *P < *0.001) and remission definitions (cCR HR: 0.18; 95% CI: 0.13, 0.23; *P < *0.001; DORIS 2021 Remission HR: 0.19; 95% CI: 0.14, 0.24; *P < *0.001; cCR-0 HR: 0.17; 95% CI: 0.13, 0.23; *P < *0.001) significantly reduced the hazards of severe flare (Table 4).

**Table 4.** Multivariable PWP gap-time models exploring the impact of target attainment on severe flare

	LDA definitions				Remission definitions					
	cLLDAS		LLDAS		cCR		DORIS 2021 Remission		cCR-0	
	HR (95% CI)	*P*-value	HR (95% CI)	*P*-value	HR (95% CI)	*P*-value	HR (95% CI)	*P*-value	HR (95% CI)	*P*-value
Disease duration (>1 year)	0.83 (0.77, 0.88)	**<0.001**	0.83 (0.77, 0.88)	**<0.001**	0.83 (0.77, 0.88)	**<0.001**	0.83 (0.77, 0.88)	**<0.001**	0.83 (0.77, 0.89)	**<0.001**
Ethnicity										
Asian	0.83 (0.68, 1.00)	**0.045**	0.83 (0.69, 1.00)	**0.047**	0.83 (0.69, 1.01)	0.062	0.84 (0.69, 1.01)	0.068	0.81 (0.67, 0.99)	**0.037**
White British	0.83 (0.70, 0.99)	**0.041**	0.84 (0.70, 1.00)	**0.047**	0.83 (0.70, 1.00)	**0.048**	0.84 (0.70, 1.00)	0.050	0.82 (0.68, 0.98)	**0.030**
Target state attainment at any time point	0.18 (0.14, 0.23)	**<0.001**	0.18 (0.14, 0.24)	**<0.001**	0.18 (0.13, 0.23)	**<0.001**	0.19 (0.14, 0.24)	**<0.001**	0.17 (0.13, 0.23)	**<0.001**
Increasing SDI score during follow-up	1.09 (1.05, 1.14)	**<0.001**	1.09 (1.05, 1.14)	**<0.001**	1.10 (1.05, 1.14)	**<0.001**	1.10 (1.06, 1.14)	**<0.001**	1.09 (1.04, 1.14)	**<0.001**

Hazard ratios (HRs) and 95% CIs are reported. cCR: cSLE clinical remission on steroids; cCR-0: cSLE clinical remission off steroids; cLLDAS: childhood lupus low disease activity state; DORIS 2021 Remission: single definition of remission proposed by the DORIS Task Force in 2021; LDA: low disease activity; LLDAS: lupus low disease activity state; PWP: Prentice–Williams–Peterson; SDI: SLICC Standardized Damage Index.

On comparing the HRs for target attainment pairwise between paediatric- and adult-specific targets, no statistically significant differences were found (*P >* 0.05, Supplementary Table S6, available at *Rheumatology* online), suggesting comparability in their ability to reduce severe flares.

### Impact of target attainment on new damage

The univariable PWP gap-time models evaluating the impact of target attainment on new damage (Supplementary Table S4, available at *Rheumatology* online) demonstrated that meeting any of the LDA target definitions (cLLDAS HR: 0.22; 95% CI: 0.11, 0.44; *P < *0.001; LLDAS HR: 0.24; 95% CI: 0.13, 0.46; *P < *0.001) and remission targets (cCR HR: 0.25; 95% CI: 0.13, 0.49; *P < *0.001; DORIS 2021 Remission HR: 0.27; 95% CI: 0.14, 0.50; *P < *0.001; cCR-0 HR: 0.30; 95% CI: 0.15, 0.60; *P < *0.001) significantly reduced the hazards of new damage.

Statistical pairwise comparisons between corresponding paediatric- and adult-specific targets highlighted no significant difference between the HRs (*P >* 0.05, Supplementary Table S6, available at *Rheumatology* online), signifying comparable protective effects from new damage.

## Discussion

This study validates novel T2T endpoints for cSLE [31], assessing their attainability and impact on severe flares and new damage prevention in a prospective cSLE cohort. We demonstrated that cSLE and aSLE T2T endpoints were similarly attainable and effective in reducing severe flares and new damage. The introduction of a weight-based prednisolone cut-off in cSLE targets (cLLDAS and cCR) prevented misclassification of disease control in younger, lighter cSLE patients. Whilst long-term corticosteroid use may control cSLE disease activity, it causes burdensome side effects, particularly in childhood [39]. Therefore, incorporating cSLE-specific T2T targets and ultimately aiming for cCR-0 is essential to optimize treatment and avoid excessive corticosteroid use.

These data demonstrate some difference in the attainment of aSLE and cSLE targets, with cSLE targets being reached at a lower number of visits due to patients being on inappropriately high weight-based corticosteroid dosages. The longer time to reach paediatric targets also reflects the requirement to adjust corticosteroid doses to the suitable weight-based cut-off threshold. Patients meeting only adult-specific targets (and not cSLE targets) were younger and lighter, suggesting misclassification of disease control, with lighter patients receiving disproportionately high prednisolone doses. These findings affirm that the slightly longer timeframe and stricter criteria for paediatric targets align with the goal of minimizing corticosteroid exposure in lighter/younger cSLE patients [14].

All cSLE T2T endpoints reduced risk of severe flares and new damage, as previously documented [17]. Notably, paediatric- and adult-specific targets demonstrated comparable protection against severe flares and new damage. However, the HR for severe flares with cCR *vs* DORIS 2021 Remission was close to significance (*P*-value of 0.052), and therefore larger studies would be useful to assess if a statistically meaningful difference can be detected. Additionally, the study highlighted lower flare risk for White British and Asian patients compared with African/Caribbean patients, supporting previous findings showing higher disease activity in African populations [40–42]. These models also highlighted the critical nature of the first year, as evident by the reduced severe flare risks when disease duration exceeds 1 year [43]. During this initial year following diagnosis, patients face the most severe disease manifestations [44], highest standardized mortality rate [45] and most intensive corticosteroid treatment [43]. A UK and Republic of Ireland cSLE epidemiological study emphasized that nearly all patients required systemic corticosteroid treatment (98%), and a significant proportion (20.4%) experienced damage accrual during the first year of the disease [43]. These data reinforce the value of T2T strategies, optimization of immunosuppression to reach the target dose in a growing child, with the aim of controlling disease activity and improving outcomes.

Presence of high anti-dsDNA antibodies emerged as a predictor for spending less time in cLLDAS and LLDAS during follow-up. This is to be expected, as anti-dsDNA positivity contributes to the SLEDAI-2K score, and is often correlated with higher disease activity [46]. Low C3 levels were found to also be a predictor of spending less time in all paediatric- and adult-specific targets. This was somewhat unexpected since the cSLEDAI score, used by the remission targets, does not account for complement levels. However, low C3 is widely known to be associated with increased disease activity and immune dysregulation [47, 48].

This study has some limitations. First, it did not consider mutual exclusiveness of target definitions, as patients moved between target states during follow-up. The sample size was also smaller compared with aSLE validation studies, with a low number of new damage events in the cCR-0 group, affecting the statistical power in particular. UK JSLE Cohort Study data are collected alongside routine clinical practice, necessitating imputation for missing data, such as patients’ weights. Corticosteroid toxicity was not recorded, so the impact of target attainment on corticosteroid toxicity remains undetermined. Future studies could utilize the recently developed paediatric Glucocorticoid Toxicity Index (pGTI) [49] to explore this issue further. This study relies on real-world data, which can potentially introduce biases, including selection bias from patient exclusions and information bias from imputing missing data. Loss to follow-up may lead to selectively including patients more likely to remain engaged, potentially affecting generalizability. Imputation of missing data, while necessary, may introduce inaccuracies that could influence the findings. Future research should involve larger, international cohorts and additional clinical trial-based validation of cSLE T2T endpoints to address these biases.

## Conclusions

This study validated novel cSLE T2T endpoints using a large national cSLE cohort, demonstrating that cSLE and aSLE targets are comparably attainable and effective in mitigating severe flares and new damage. It highlighted that paediatric modifications prevent underestimation of disease activity in younger, lighter cSLE patients. With careful monitoring, cSLE patients can anticipate improved outcomes and minimize their corticosteroid exposure. Future research should explore the implementation of target-driven care and compare the outcomes of target-driven care with standard care.

## Supplementary Material

keaf057_Supplementary_Data

## Data Availability

Data will be made available on reasonable request.
