# Supplementary material for: Validation of childhood lupus specific targets: ensuring accurate assessment of disease control in younger, lighter paediatric patients
Source: Rheumatology (Oxford). 2025 Feb 6;64(6):3587–97. doi: 10.1093/rheumatology/keaf057 (PMC12107070; doi:10.1093/rheumatology/keaf057)
Supplement: keaf057_Supplementary_Data [file keaf057_supplementary_data.docx]

**Supplementary material**

**Supplementary Table S1. Factors explored in univariable logistic regression models and PWP gap-time models.**

| Description of analysis conducted in study | Factors that were explored in the analysis |
| --- | --- |
| Univariable logistic regression exploring factors associated with spending a greater length of cumulative follow-up time in target | - Low C3 - Low C4 - Anti-dsDNA positivity - Low platelets - Low haemoglobin - Leucopaenia - Lymphopaenia - Proteinuria - ESR - BILAG-defined organ-domain involvement (constitutional, mucocutaneous, neuropsychiatric, musculoskeletal, cardiorespiratory, gastrointestinal, ophthalmic, renal, haematological) - BILAG numerical score - Sex - Ethnicity - Age at diagnosis - SLICC-SDI score - SLEDAI-2K score |
| Univariable PWP gap-time models exploring the impact of target attainment, clinical and demographic factors on severe flare and new damage | - Sex - Disease duration - Ethnicity - SDI score of ≥ 1 at study recruitment - Increasing SDI score during follow-up - Target state attainment at any time point (cLLDAS, LLDAS, cCR, DORIS 2021 Remission, cCR-0) |
| Anti-dsDNA positive, anti-double stranded DNA antibody positivity; BILAG, British Isles Lupus Assessment Group; cCR, cSLE clinical remission on steroids; cCR-0, cSLE clinical remission off steroids; cLLDAS, childhood lupus low disease activity state; C3/C4, complement factor 3/4; DORIS 2021 Remission, single definition of Remission proposed by the DORIS Task Force in 2021; ESR, erythrocyte sedimentation rate; LLDAS, lupus low disease activity state; PWP, Prentice-Williams-Peterson; SLICC-SDI, Systemic Lupus International Collaborating Clinics Standardised Damage Index; SLEDAI-2K, systemic lupus erythematosus disease activity index. | |

**Supplementary Table S2. Wilcoxon signed-rank/rank-sum tests exploring the comparisons between targets from descriptive analyses.**

|  | Paediatric- vs adult-specific targets* | | Among paediatric-specific targets** | | |
| --- | --- | --- | --- | --- | --- |
|  | **cLLDAS vs LLDAS** | **cCR vs DORIS 2021 Remission** | **cLLDAS vs cCR** | **cLLDAS vs cCR-0** | **cCR vs cCR-0** |
| Time to target attainment | **<0.001** | **<0.001** | **<0.001** | **<0.001** | **<0.001** |
| Percentage of time in target per patient | 0.986 | 0.998 | 0.871 | 0.597 | 0.768 |
| Length of time in target | 0.996 | 0.972 | 0.968 | 0.947 | 0.947 |
| The *p*-values from Wilcoxon signed-rank/rank-sum tests are reported. Bonferroni correction was applied for multiple comparison testing by adjusting the *p*-value level threshold for significance (**p*<0.03, ***p*<0.02). cCR, cSLE clinical remission on steroids; cCR-0, cSLE clinical remission off steroids; cLLDAS, childhood lupus low disease activity state; DORIS 2021 Remission, single definition of Remission proposed by the DORIS Task Force in 2021. | | | | | |

**Supplementary Table S3. Univariable logistic regression exploring factors associated with spending a greater length of cumulative follow-up time in target.**

|  | cLLDAS | | LLDAS | | cCR | | DORIS 2021 Remission | | cCR-0 | |
| --- | --- | --- | --- | --- | --- | --- | --- | --- | --- | --- |
|  | **OR (95% CI)** | ***p*-value** | **OR (95% CI)** | ***p*-value** | **OR (95% CI)** | ***p*-value** | **OR (95% CI)** | ***p*-value** | **OR (95% CI)** | ***p*-value** |
| Low C3 (<1.04 g/l) | 0.45  (0.28, 0.75) | **0.002** | 0.43  (0.26, 0.70) | **<0.001** | 0.43  (0.26, 0.73) | **0.001** | 0.47 (0.28, 0.78) | **0.004** | 0.37  (0.21,0.65) | **<0.001** |
| Low C4 (<0.18 g/l) | 0.57  (0.33, 0.96) | **0.034** | 0.52  (0.31, 0.88) | **0.014** | 0.51  (0.30, 0.88) | **0.016** | 0.55 (0.32, 0.94) | **0.030** | 0.58  (0.32, 1.06) | 0.078 |
| Anti-dsDNA positive (>20) | 0.41  (0.24, 0.70) | **0.001** | 0.38  (0.22, 0.64) | **<0.001** | 0.68 (0.39, 1.18) | 0.171 | 0.63  (0.37, 1.09) | 0.099 | 0.57  (0.31, 1.04) | 0.068 |
| Low platelets (<100 10^9^/l) | 0.83  (0.37, 1.83) | 0.641 | 0.79  (0.36, 1.76) | 0.574 | 1.39 (0.65, 2.96) | 0.395 | 1.52  (0.72, 3.18) | 0.270 | 1.74  (0.80, 3.81) | 0.164 |
| Leucopaenia (<4x10^9^/l) | 0.82  (0.49, 1.36) | 0.441 | 0.83  (0.50, 1.38) | 0.483 | 0.91 (0.54, 1.54) | 0.728 | 0.85  (0.50, 1.44) | 0.546 | 0.83  (0.46, 1.50) | 0.537 |
| Low haemoglobin (<9 g/dl) | 0.82  (0.43, 1.53) | 0.527 | 0.96  (0.52, 1.76) | 0.888 | 0.65 (0.32, 1.30) | 0.222 | 0.95  (0.50, 1.79) | 0.876 | 0.85  (0.41, 1.75) | 0.652 |
| Lymphopaenia (<1.5x10^9^/l) | 0.89  (0.57, 1.38) | 0.598 | 0.88  (0.57, 1.36) | 0.570 | 0.80 (0.51, 1.26) | 0.340 | 0.82  (0.52, 1.28) | 0.377 | 0.56  (0.34, 0.92) | **0.024** |
| Proteinuria (>50 mg or dipstick = 3) | 0.92  (0.54, 1.55) | 0.751 | 0.88  (0.52, 1.49) | 0.636 | 1.18  (0.69, 2.01) | 0.542 | 1.09  (0.64, 1.85) | 0.743 | 0.69  (0.36, 1.31) | 0.250 |
| ESR | | | | | | | | | | |
| ≤50 mm/hr | 0.64  (0.34, 1.19) | 0.158 | 0.69  (0.37, 1.28) | 0.240 | 0.89 (0.47, 1.68) | 0.722 | 0.93  (0.49, 1.74) | 0.810 | 0.69  (0.35, 1.37) | 0.286 |
| >50 mm/hr | 0.76  (0.41, 1.39) | 0.376 | 0.79  (0.43, 1.44) | 0.438 | 0.73 (0.38, 1.38) | 0.330 | 0.79  (0.42, 1.49) | 0.461 | 0.60  (0.30, 1.19) | 0.144 |
| BILAG defined organ-domain involvement | | | | | | | | | | |
| Constitutional | 1.05  (0.67, 1.65) | 0.834 | 1.05  (0.67, 1.65) | 0.819 | 1.19 (0.75, 1.90) | 0.462 | 1.15  (0.73, 1.82) | 0.547 | 0.91  (0.54, 1.53) | 0.712 |
| Mucocutaneous | 0.89  (0.58, 1.37) | 0.589 | 0.88  (0.58, 1.36) | 0.572 | 0.95 (0.60, 1.48) | 0.807 | 0.95  (0.61, 1.48) | 0.831 | 1.09  (0.67, 1.78) | 0.734 |
| Neuropsychiatric | 0.84  (0.41, 1.73) | 0.641 | 0.93  (0.46, 1.87) | 0.832 | 1.20 (0.59, 2.44) | 0.614 | 1.27  (0.64, 2.55) | 0.493 | 0.81  (0.35, 1.89) | 0.622 |
| Musculoskeletal | 0.75  (0.47, 1.20) | 0.227 | 0.68  (0.42, 1.08) | 0.103 | 0.93 (0.58, 1.51) | 0.772 | 0.91  (0.57, 1.46) | 0.689 | 0.81  (0.48, 1.39) | 0.454 |
| Cardiorespiratory | 0.83  (0.43, 1.62) | 0.586 | 0.80  (0.41, 1.56) | 0.512 | 1.04 (0.53, 2.04) | 0.910 | 1.09  (0.56, 2.10) | 0.800 | 0.76  (0.34, 1.69) | 0.504 |
| Gastrointestinal | 1.43  (0.55, 3.72) | 0.462 | 1.38  (0.53, 3.59) | 0.507 | 2.23 (0.87, 5.70) | 0.0934 | 2.09  (0.82, 5.34) | 0.123 | 1.50  (0.53, 4.29) | 0.449 |
| Ophthalmic | 7.39  (0.76, 71.7) | 0.0846 | 7.14  (0.74, 69.3) | 0.090 | 9.06 (0.93, 88.0) | 0.0575 | 8.51  (0.88, 82.7) | 0.065 | 1.38  (0.14, 13.4) | 0.783 |
| Renal | 0.97  (0.62, 1.51) | 0.891 | 0.97  (0.63, 1.51) | 0.893 | 1.18 (0.75, 1.86) | 0.479 | 1.20  (0.76, 1.87) | 0.436 | 1.02  (0.61, 1.69) | 0.946 |
| Haematological | 0.99  (0.61, 1.61) | 0.964 | 1.07  (0.66, 1.73) | 0.786 | 0.81 (0.48, 1.36) | 0.420 | 0.98  (0.59, 1.62) | 0.937 | 0.84  (0.47, 1.49) | 0.546 |
| BILAG numerical score | 1.00  (0.98, 1.02) | 0.991 | 1.00  (0.98, 1.02) | 0.949 | 1.01 (0.98, 1.03) | 0.503 | 1.01  (0.99, 1.03) | 0.427 | 0.99  (0.96, 1.02) | 0.425 |
| Sex  Male* | 1.50  (0.88, 2.56) | 0.140 | 1.55  (0.91, 2.63) | 0.108 | 1.92 (1.12, 3.30) | **0.018** | 1.92  (1.12, 3.28) | **0.017** | 2.15  (1.21, 3.81) | **0.009** |
| Ethnicity** | | | | | | | | | | |
| Asian | 1.79  (0.91, 3.49) | 0.089 | 2.01  (1.02, 3.97) | **0.044** | 2.04 (0.96, 4.33) | 0.0646 | 1.98  (0.95, 4.13) | 0.067 | 1.66  (0.72, 3.79) | 0.232 |
| White British | 1.37  (0.73, 2.58) | 0.330 | 1.59  (0.84, 3.04) | 0.155 | 1.99 (0.98, 4.06) | 0.0585 | 1.88  (0.94, 3.75) | 0.074 | 1.90  (0.88, 4.12) | 0.103 |
| Diagnosis Age | 0.98  (0.92, 1.05) | 0.599 | 0.96  (0.90, 1.02) | 0.156 | 0.96 (0.90, 1.03) | 0.240 | 0.94  (0.88, 1.01) | 0.078 | 0.93  (0.87, 1.00) | **0.040** |
| SLICC-SDI score | | | | | | | | | | |
| Mild | 0.62  (0.30, 1.25) | 0.180 | 0.67  (0.34, 1.34) | 0.258 | 1.45 (0.76, 2.77) | 0.255 | 1.50  (0.79, 2.83) | 0.212 | 1.02  (0.49, 2.15) | 0.954 |
| Moderate | 0.61  (0.12, 2.97) | 0.539 | 0.59  (0.12, 2.90) | 0.517 | 0.38 (0.05, 3.04) | 0.358 | 0.35  (0.04, 2.86) | 0.329 | 0.50  (0.06, 4.05) | 0.515 |
| Severe | 0.71  (0.14, 3.57) | 0.677 | 0.69  (0.14, 3.48) | 0.653 | 1.00 (0.20, 5.05) | 1.00 | 0.94  (0.19, 4.75) | 0.941 | 0.57  (0.07, 4.70) | 0.601 |
| SLEDAI score | 0.98  (0.95, 1.01) | 0.112 | 0.97  (0.95, 1.00) | 0.085 | 1.00 (0.97, 1.03) | 0.918 | 1.00  (0.97, 1.03) | 0.841 | 0.97  (0.94, 1.01) | 0.102 |
| Odds ratios (ORs) and 95% confidence intervals (95% CIs) are reported. *Reference for sex are females. **Reference for ethnicity are African/Caribbean patients. Anti-dsDNA positive, anti-double stranded DNA antibody positivity; BILAG, British Isles Lupus Assessment Group; cCR, cSLE clinical remission on steroids; cCR-0, cSLE clinical remission off steroids; cLLDAS, childhood lupus low disease activity state; C3/C4, complement factor 3/4; DORIS 2021 Remission, single definition of Remission proposed by DORIS Task Force in 2021; ESR, erythrocyte sedimentation rate; LLDAS, lupus low disease activity state; SLICC-SDI, Systemic Lupus International Collaborating Clinics Standardised Damage Index; SLEDAI-2K, systemic lupus erythematosus disease activity index. | | | | | | | | | | |

**Supplementary Table S4. Impact of target attainment, clinical and demographic characteristics on severe flare and new damage.**

|  | Severe flare | | New damage | |
| --- | --- | --- | --- | --- |
|  | **HR (95% CI)** | ***p*-value** | **HR (95% CI)** | ***p*-value** |
| Sex (female) | 0.99  (0.80, 1.21) | 0.895 | 1.18  (0.70, 2.00) | 0.532 |
| Disease duration (> 1 year) | 0.80  (0.74, 0.86) | **<0.001** | 0.95  (0.83, 1.09) | 0.456 |
| Ethnicity | | | | |
| Asian | 0.78  (0.62, 0.98) | **0.035** | 0.91  (0.53, 1.56) | 0.732 |
| White British | 0.79  (0.64, 0.97) | **0.027** | 0.74  (0.44, 1.25) | 0.259 |
| SDI score of ≥ 1 at study recruitment | 1.13  (1.02, 1.25) | **0.015** | NA1 | NA1 |
| Increasing SDI score during follow-up | 1.10  (1.04, 1.17) | **<0.001** | NA1 | NA1 |
| Target state attainment at any time point | | | | |
| cLLDAS | 0.17  (0.13, 0.22) | **<0.001** | 0.22  (0.11, 0.44) | **<0.001** |
| LLDAS | 0.17  (0.13, 0.22) | **<0.001** | 0.24  (0.13, 0.46) | **<0.001** |
| cCR | 0.16  (0.12, 0.21) | **<0.001** | 0.25  (0.13, 0.49) | **<0.001** |
| DORIS 2021 Remission | 0.17  (0.13, 0.22) | **<0.001** | 0.27  (0.14, 0.50) | **<0.001** |
| cCR-0 | 0.15  (0.11, 0.21) | **<0.001** | 0.30*  (0.15, 0.60) | **<0.001** |
| Univariable PWP models were conducted, reporting the hazard ratios (HRs) and 95% confidence intervals (95% CIs). *Only 8 patients accrued damage during follow-up when attaining cCR-0 influencing the reliability of this result due to low sample size. cCR, cSLE clinical remission on steroids; cCR-0, cSLE clinical remission off steroids; cLLDAS, childhood lupus low disease activity state; DORIS 2021 Remission, single definition of Remission proposed by the DORIS Task Force in 2021; LLDAS, lupus low disease activity state; PWP, Prentice-Williams-Peterson; SDI, SLICC standardised damage index; NA1, modelling was not possible as SDI score was utilised to define new damage. | | | | |

**Supplementary Table S5. Risk of severe flare with respect to increasing percentage of cumulative follow-up time in target.**

|  | 10% | 20% | 40% | 50% | 60% | 80% |
| --- | --- | --- | --- | --- | --- | --- |
| cLLDAS | 0.69 | 0.47 | 0.22 | 0.15 | 0.11 | 0.05 |
| LLDAS | 0.69 | 0.48 | 0.23 | 0.16 | 0.11 | 0.05 |
| cCR | 0.70 | 0.49 | 0.24 | 0.17 | 0.12 | 0.06 |
| DORIS 2021 Remission | 0.71 | 0.50 | 0.25 | 0.18 | 0.12 | 0.06 |
| cCR-0 | 0.69 | 0.47 | 0.22 | 0.15 | 0.11 | 0.05 |
| Hazard ratios are computed to represent risk of severe flare. cCR, cSLE clinical remission on steroids; cCR-0, cSLE clinical remission off steroids; cLLDAS, childhood lupus low disease activity state; DORIS 2021 Remission, single definition of Remission proposed by the DORIS Task Force in 2021; LLDAS, lupus low disease activity state. | | | | | | |

|  | Severe flare | | |
| --- | --- | --- | --- |
|  | **Target 1 achieved** | **Target 2 achieved** | ***p*-value*** |
| LDA definitions | LLDAS | cLLDAS | 0.178 |
| Remission definitions | DORIS 2021 Remission | cCr | 0.052 |
|  | **New damage** | | |
|  | **Target 1 achieved** | **Target 2 achieved** | ***p*-value*** |
| LDA definitions | LLDAS | cLLDAS | 0.135 |
| Remission definitions | DORIS 2021 Remission | cCr | 0.309 |
| The hazard ratios (HRs) from univariable and multivariable PWP gap-time models were compared between paediatric- and adult-specific targets for new damage and severe flare, respectively. Bonferroni correction was applied for multiple comparison testing by adjusting the *p*-value level threshold for significance (**p*<0.03). cCR, cSLE clinical remission on steroids; cLLDAS, childhood lupus low disease activity state; DORIS 2021 Remission, single definition of Remission proposed by the DORIS Task Force in 2021; LLDAS, lupus low disease activity state; PWP, Prentice-Williams-Peterson. | | | |

**Supplementary Table S6. Two-sided t-tests comparing HRs of paediatric-specific and adult-specific targets for severe flare/new damage outcomes.**
